# Serotypes and antimicrobial susceptibility of *Salmonella* spp. isolated from farm animals in China

**DOI:** 10.3389/fmicb.2015.00602

**Published:** 2015-06-22

**Authors:** Xiuhua Kuang, Haihong Hao, Menghong Dai, Yulian Wang, Ijaz Ahmad, Zhenli Liu, Yuan Zonghui

**Affiliations:** ^1^MOA Laboratory for Risk Assessment of Quality and Safety of Livestock and Poultry Products, Huazhong Agricultural UniversityWuhan, China; ^2^Hubei Collaborative Innovation Center for Animal Nutrition and Feed Safety, Huazhong Agricultural UniversityWuhan, China; ^3^National Reference Laboratory of Veterinary Drug Residues and MOA Key Laboratory for Detection of Veterinary Drug Residues, Huazhong Agricultural UniversityWuhan, China

**Keywords:** *Salmonella*, farm animals, serotype, antibiotics, antimicrobial resistance

## Abstract

*Salmonella* spp. can indirectly infect humans via transfer from animals and animal-derived food products, and thereby cause potentially fatal diseases. Therefore, gaining an understanding of *Salmonella* infection in farm animals is increasingly important. The aim of this study was to identify the distribution of serotypes in *Salmonella* samples isolated from chickens (*n* = 837), pigs (*n* = 930), and dairy cows (*n* = 418) in central China (Henan, Hubei, and Hunan provinces) in 2010–2011, and investigate the susceptibility of strains to antimicrobial agents. *Salmonella* isolates were identified by PCR amplification of the *invA* gene, serotypes were determined by using a slide agglutination test for O and H antigens, and susceptibility to 24 antimicrobials was tested using the agar dilution method. In total, 248 *Salmonella* strains were identified: 105, 105, and 38 from chickens, dairy cows, and pigs, respectively. Additionally, 209 strains were identified in diseased pigs from the Huazhong Agricultural University veterinary hospital. Among these 457 strains, the dominant serotypes were Typhimurium in serogroup B, IIIb in serogroup C, and Enteritidis in serogroup D. In antimicrobial susceptibility tests, 41.14% of *Salmonella* spp. were susceptible to all antimicrobial agents, 48.14% were resistant to at least one, and 34.72% were resistant to more than three classes. Strains were highly resistant to sulfamethoxazole-trimethoprim (39.61%), nalidixic acid (39.17%), doxycycline (28.22%), and tetracycline (27.58%). Resistance to cephalosporins and fluoroquinolones ranged from 5.25 to 7.44% and 19.04 to 24.51%, respectively. Among penicillin-resistant and cephalosporin-resistant strains, 25 isolates produced extended-spectrum β-lactamases (ESBLs). The multidrug-resistant and ESBL-producing *Salmonella* strains identified in healthy animals here will present a challenge for veterinary medicine and farm animal husbandry, and could also pose a threat to public health. The level of antibiotic resistance observed in this study further highlights the need for careful and selective use of antibiotics.

## Introduction

*Salmonella* spp. are a common source of foodborne diseases that cause morbidity and mortality worldwide (Chiu et al., 2010). Treating *Salmonella* infection in humans is expensive; for example, in the USA, it causes illness in ~1.2 million patients annually, resulting in estimated medical costs of $365 million (Centers for Disease Control and Prevention, 2014). In the European Union (EU), salmonellosis is the second most commonly reported gastrointestinal infection, with a confirmed case rate of 20.4 cases per 100,000 individuals in 2011 (European Centre for Disease Prevention and Control, 2013). In China, *Salmonella* causes an estimated 22.2% of foodborne diseases (Wang et al., 2007), and salmonellosis ranks fourth among the most prevalent foodborne diseases caused by microbial agents (Zhu et al., 2012). Many *Salmonella* serotypes exist, with >2600 serovars classified based on the reactivity of antisera to O and H antigens (Stevens et al., 2009), and ~292 identified in China between the 1980s and the end of 2000 (Yang, 2010). *Salmonella* also affects farm animals, and infections on farms can cause substantial economic damage in relation to, for example, loss of poultry stocks and costly animal husbandry. Numerous serotypes of isolated *Salmonella* have been found to overlap between farm animals and humans (Alcaine et al., 2006). Indeed, *Salmonella* not only directly infects humans but also causes indirect infections via transfer from animals and animal-derived food products such as pork and milk.

The use of antimicrobials is important for the control and treatment of *Salmonella*. However, since the early 1990s, antimicrobial- and multidrug-resistant *Salmonella* strains have emerged, leading to treatment failure. Researchers have reported a link between the use of antimicrobials in food animals and the emergence of antimicrobial resistance in pathogenic bacteria (Gong et al., 2013). Multidrug-resistant bacteria pose a severe threat to public health, particularly those that are resistant to β-lactams and fluoroquinolones (Lai et al., 2014). The increasing number of multidrug-resistant *Salmonella* strains is a global concern, with some countries and international organizations creating surveillance systems which include collaboration between human health, veterinary, and food-related sectors to monitor the spread of these and other foodborne bacteria. Examples include the Danish Integrated Antimicrobial Resistance Monitoring and Research Program, the European Food Safety Authority, the National Antimicrobial Resistance Monitoring System in the USA, and the Global Foodborne Infections Network run by the World Health Organization. These surveillance systems are also employed to monitor antimicrobial resistance, antimicrobial consumption in livestock, and serotype distribution, and data describing the current trend of increasing resistance to multiple drugs has been made available (European Centre for Disease Prevention and Control, 2013). In contrast, surveillance reports are unavailable in China; however, the National Monitoring Network for Bacteria of Animal Origin was launched as a surveillance system in 2008. Travel, migration, and the distribution of food between countries can also contribute to the spread of foodborne diseases and multidrug-resistant bacteria. Therefore, monitoring the distribution of *Salmonella* serotypes and levels of antibiotic resistance in animals and animal-food products is also important for maintaining safe travel and the commercial trade in food animals (Lai et al., 2014; Russell et al., 2014).

In central China, Henan, Hubei, and Hunan provinces are important residential and trade centers. These three areas are also the main producers of animal-derived foods in China; in 2012, their output of meat, eggs, and milk accounted for 19.14%, 19.45%, and 9.17% of total Chinese production, respectively (China Agriculture Statistical Report, 2012). Previous studies documented a phenotype for *Salmonella* serovars that was discovered in chickens and pigs, and in animal-derived foods, in China (Gong et al., 2013; Li et al., 2013; Lai et al., 2014; Wang et al., 2014). However, specific knowledge of the *Salmonella* serovars that exist in dairy cows and other farm animals in central China is currently lacking. Therefore, the aim of this study was to evaluate the prevalence of *Salmonella* in chickens, pigs, and dairy cows on farms in central China. Additionally, the diversity of *Salmonella* serovars on these farms was identified, and their susceptibility and resistance to antimicrobial agents was investigated. A diverse range of serotypes was observed in healthy and diseased farm animals, and several multidrug-resistant and extended-spectrum β-lactamase (ESBL)-producing *Salmonella* strains were identified.

## Materials and methods

### Samples and *Salmonella* isolation

Cloacal and anal swabs were collected from healthy animals on farms in Henan, Hubei, and Hunan provinces between March 2010 and July 2011. Each sampling site was visited only once. In total, 2185 samples were collected in a random manner from chickens (*n* = 837), pigs (*n* = 930), and dairy cows (*n* = 418). Farms were chosen based on their scale with the following requirements: for pigs, annual sales were >10,000 heads; for chickens, the breeding stock was >100,000 heads; and for dairy cows, the breeding stock was >1000 heads. The owners of each farm gave permission for swab samples to be to collected. The animals from which samples were extracted remained alive, did not undergo any surgery, and were not administered any drugs. Therefore, ethical approval was not required for the study because the sampling process did not harm the animals; however, their distress was considered and minimized at all times. All collected samples were stored at 4 °C and cultured at least 24 h before the isolation experiments were conducted.

The procedure for culture and isolation was based on standard laboratory protocol (WHO Global Foodborne Infections Network, 2010). Briefly, the samples were mixed with 5 mL of 0.85% saline solution for 30 min, and 1 mL of this mixture solution was added to buffered peptone water (BPW; Hopebiol, Qingdao, China) at a volume ratio of 1:10. The resultant mixture was incubated for 10 h at 37 °C for pre-enrichment. Approximately 500 μL of this BPW mixture was then added to 5 mL selenite cystine broth (Hopebiol, Qingdao, China) and incubated at 37 °C for 20 h. The broth was streaked onto CHROMagar *Salmonella* (CHROMagar, France) and incubated at 37 °C for 24–48 h. Potential *Salmonella* colonies were transferred into Luria–Bertani agar for purification and enrichment, and then incubated for 20 h at 37 °C to facilitate identification.

Additionally, 209 strains of *Salmonella* were isolated from samples collected from diseased pigs in the Huazhong Agricultural University veterinary hospital (HAUvh) between 2008 and 2010.

### *Salmonella* identification

Biochemical testing and the *invA* gene were used to confirm the identity of isolates with typical *Salmonella* phenotypes. Biochemical testing was performed using a biochemical tube (Hangzhou Microbe Reagent Co., Ltd., China), and the results were interpreted based on Bergey's Manual of Systematic Bacteriology (Garrity et al., 2004). The *invA* gene was amplified by polymerase chain reaction (PCR) (Malorny et al., 2003). Positive results were randomly selected for sequencing. The obtained sequences were compared with the same gene registered in GenBank by using BLAST.

### Determination of *Salmonella* serogroup and serotype

The serogroup and serovars of the *Salmonella* isolates were determined by slide agglutination tests with O antigen and H antigen antiserums obtained from Lansheng, Lanzhou Institute of Biology, China. The results were interpreted according to the Kauffmann-White scheme (Grimont and Weill, 2007).

### Antimicrobial susceptibility testing

The agar dilution method with Mueller-Hinton agar (Oxoid Ltd., England) was used to test the susceptibility of *Salmonella* to 24 antibiotics (Table 1). These antimicrobials were classified based on their importance to human medicine (Government of Canada, 2005). *Escherichia coli* ATCC 25922 was used as the control microorganism. The results of the tests for ampicillin (AMP), amoxicillin (AMX), amoxicillin-clavulanic acid (AMC), ceftriaxone (CRO), imipenem (IPM), aztreonam (ATM), gentamicin (GEN), amikacin (AMK), tetracycline (TET), doxycycline (DOX), ciprofloxacin (CIP), levofloxacin (LEV), nalidixic acid (NAL), sulfamethoxazole-trimethoprim (SXT), chloramphenicol (CHL), and fosfomycin (FOS) were interpreted based on CLSI M100-S22 (Clinical and Laboratory Standards Institute, 2012), whereas those for ceftiofur (CEF), enrofloxacin (ENO), and florfenicol (FFC) were interpreted based on CLSI M31-A3 (Clinical and Laboratory Standards Institute, 2008). Interpretive CLSI criteria were not available for cefquinome (CEQ), polymyxin B (PB), azithromycin (AZM), olaquindox (OLA), and mequindox (MEQ); therefore, results with the following minimum inhibitory concentration (MIC) values were considered resistant for these antibiotics: CEQ ≥8 μg/mL (using CEF as a reference); PB ≥4 μg/mL (Kwa et al., 2007); AZM ≥16 μg/mL (Sjölund-Karlsson et al., 2011); OLA and MEQ ≥64 μg/mL (Sørensen et al., 2003).

**Table 1:** **Antibiotics and the range of concentrations tested**.

**Antibiotic**	**Abbreviation**	**Concentration range (μg/mL)**
Ampicillin	AMP	0.06 ~ 256
Amoxicillin	AMX	0.06 ~ 256
Amoxicillin-clavulanic acid	AMC	0.06/0.03 ~ 128/256
Ceftriaxone	CRO	0.06 ~ 512
Ceftiofur	CEF	0.06 ~ 512
Cefquinome	CEQ	0.015 ~ 256
Imipenem	IPM	0.03 ~ 128
Aztreonam	ATM	0.06 ~ 512
Gentamicin	GEN	0.5 ~ 512
Amikacin	AMK	0.5 ~ 512
Tetracycline	TET	0.5 ~ 512
Doxycycline	DOX	0.5 ~ 512
Ciprofloxacin	CIP	0.015 ~ 512
Enrofloxacin	ENO	0.06 ~ 512
Levofloxacin	LEV	0.06 ~ 512
Nalidixic acid	NAL	0.06 ~ 512
Sulfamethoxazole-trimethoprim	SXT	0.25/4.75 ~ 128/2432
Chloramphenicol	CHL	0.5 ~ 512
Florfenicol	FFC	0.5 ~ 512
Fosfomycin	FOS	1 ~ 2,048
Polymyxin B	PB	0.5 ~ 512
Azithromycin	AZM	0.5 ~ 512
Olaquindox	OLA	0.25 ~ 128
Mequindox	MEQ	0.25 ~ 128

Strains that were resistant to AMP, AMX, ATM, and/or CRO were examined for ESBLs by using the MIC values of ceftazidime, ceftazime-clavulanic acid, cefotaxime, and cefotaxime-clavulanic acid. Results were interpreted based on CLSI M100-S22 (Clinical and Laboratory Standards Institute, 2012), and *Klebsiella pneumoniae* ATCC 700603 and *E. coli* ATCC 25922 were used as the control organisms.

### Statistical analysis

The isolated strains were categorized as sensitive (S), intermediary (I), or resistant (R) based on the MIC values and the CLSI interpretive criteria. MIC_50_ and MIC_90_ were calculated using previously described methods (Schwarz et al., 2010), and 95% confidence intervals were calculated using SPSS 16.0 software (IBM, USA).

## Results

### *Salmonella* isolates and serotypes

In total, 248 bacterial isolates taken from healthy chickens, pigs, and dairy cows, and 209 isolates from diseased pigs, were verified as *Salmonella* spp., and 457 strains identified as serotypes were tested for antimicrobial susceptibility. In all samples taken from healthy animals, the prevalence of *Salmonella* spp. was 11.35% (248/2185). The specific prevalence of *Salmonella* spp. in chickens, pigs, and dairy cows was 12.55% (105/837), 4.09% (38/930), and 25.12% (105/418), respectively.

After serotyping, all isolates were divided into three groups (Table 2). Thirty-four isolates did not cluster for self-agglutination in sterile saline. Twenty-eight isolates could self-agglutination in sterile saline, but such activity was not observed with O antigen. The serogroups of the remaining 395 strains were determined. Eight distinct serogroups and 41 distinct serotypes were identified, as shown in Table 2. Group B (*n* = 205, 44.86%) and group C1 (*n* = 141, 30.85%) were identified as the dominant serogroups. Typhimurium 12.47% (*n* = 57) and IIIb 4.81% (*n* = 22) were the dominant serovars. Detailed serotypes could not be determined for 208 *Salmonella* isolates using the available H antigen in the different serogroups. According to the species of animal tested, the dominant serotypes were Typhimurium and Enteritidis in chickens, IIIb and Typhimurium in pigs, and Typhimurium and Agona in dairy cows (Table 2).

**Table 2:** **Serovar distribution of isolated *Salmonella* (*n* = 457)**.

**Serotype (N)^a^**	**Serovars**	**Chicken (n^b^)**	**Pig (n^b^)**	**Dairy cow (n^b^)**
Unable to self- agglutination (34)		6	28	0
Group A (3)	Unidentified types	3	0	0
Group B (205)	Unidentified subtypes	31	19	45
	Typhimurium	20	11	26
	Agona	3	0	12
	Derby	1	2	6
	II	1	1	4
	Kunduchi	0	0	4
	Schwarzenground	0	3	0
	Lagos	0	2	1
	Fyris	0	0	2
	Agama	1	1	0
	Farsta	1	0	0
	Gloucester	1	0	0
	Kingston	0	0	1
	Kubacha	1	0	0
	Saintpaul	1	0	0
	Stanley	0	1	0
	Travis	0	1	0
	Tumodi	0	0	1
	Uppsala	0	1	0
Group C1 (141)	Unidentified subtypes	0	90	1
	IIIb	0	22	0
	Typhisuis	0	7	0
	II	0	6	0
	Schwabach	0	5	0
	Irumu	0	3	0
	Kaduna	0	2	0
	Birkenhead	0	1	0
	Cotonou	0	1	0
	Hissar	0	1	0
	Paratyphic	0	1	0
	Thompson	0	1	0
Group C2-C3 (5)	Unidentified subtypes	0	4	0
	Quiniela	0	1	0
Group D1 (16)	Unidentified subtypes	4	0	0
	Enteritidis	9	0	1
	II	1	0	0
	Berta	1	0	0
Group D3 (1)	II	0	1	0
Group E1 (4)	Meleagridis	1	0	0
	Newlands	0	2	0
	Simi	1	0	0
Group E4 (20)	Unidentified subtypes	2	9	0
	Kouka	4	0	0
	Gnesta	0	3	0
	Visby	0	1	0
	Rideau	1	0	0
Unidentified subgroups (28)		11	16	1

a *N, the total number of Salmonella isolates per serogroup*.

b *n, number of isolates with a given serotype of animals*.

### Antimicrobial susceptibility testing

Results of antimicrobial susceptibility testing are summarized in Table 3. Among the 457 isolates, 188 (41.14%) were susceptible to all tested antimicrobials and 220 (48.14%) were resistant to at least one antibiotic. All strains showed susceptibility or intermediate susceptibility to imipenem. Strains were most commonly resistant to nalidixic acid (39.17%), sulfamethoxazole-trimethoprim (39.61%), doxycycline (28.22%), and tetracycline (27.58%). They also showed resistance to all specialist drugs used only in veterinary medicine, including enrofloxacin (24.51%), florfenicol (20.13%), ceftiofur (7.44%), and cefquinome (5.25%), and to drugs used in both veterinary and human medicine, such as ciprofloxacin (24.07%), gentamicin (19.69%), ceftriaxone (6.34%), and amoxicillin/clavulanate (4.38%). Resistance to azithromycin, which is one of the macrolides often used against gram-positive bacteria, was expressed at 16.19%. Although they are rarely used in clinical applications, resistance to polymyxin B (18.82%) and fosfomycin (12.04%) was also observed.

**Table 3:**
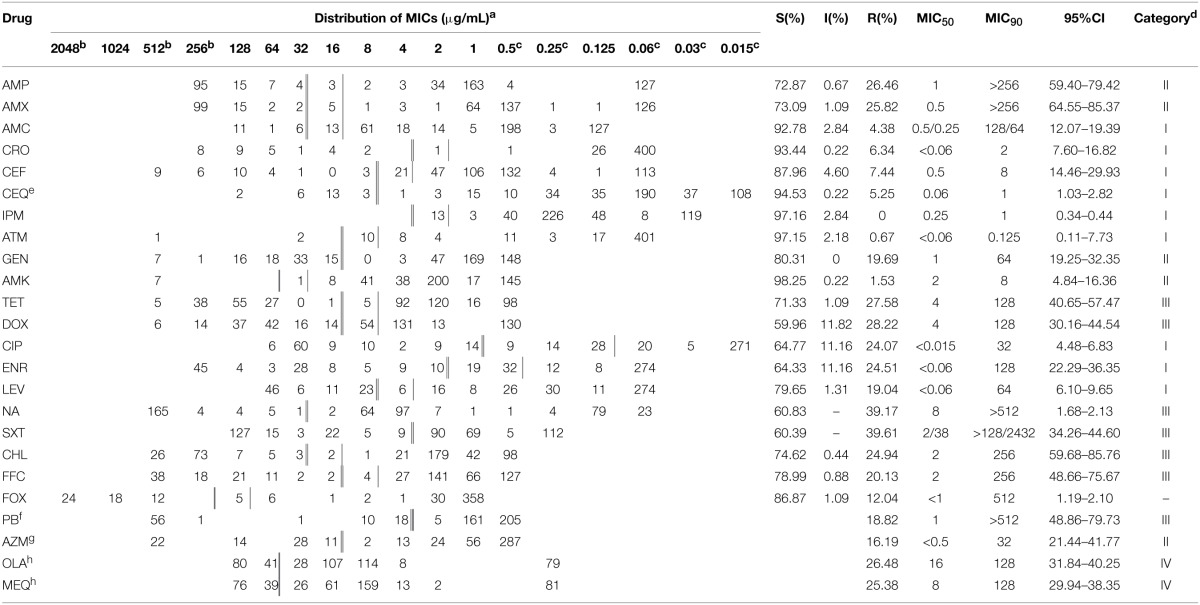
**Susceptibility of *Salmonella* isolates (*n* = 457) to antimicrobials**.

*AMP, Ampicillin; AMX, Amoxicillin; AMC, Amoxicillin-clavulanic acid; CRO, Ceftriaxone; CEF, Ceftiofur; CEQ, Cefquinome; IPM, Imipenem; ATM, Aztreonam; GEN, Gentamicin; AMK, Amikacin; TET, Tetracycline; DOX, Doxycycline; CIP, Ciprofloxacin; ENO, Enrofloxacin; LEV, Levofloxacin; NAL, Nalidixic acid; SXT, Sulfamethoxazole-trimethoprim; CHL, Chloramphenicol; FFC, Florfenicol; FOS, Fosfomycin; PB, Polymyxin B; AZM, Azithromycin; OLA, Olaquindox; MEQ, Mequindox*.

*^a^Susceptibility breakpoints are indicated by black vertical bars and resistance breakpoints are double vertical bars. CLSI breakpoints used when available. CLSI breakpoints do not exist for cefquinome, polymyxin B, azithromycin, olaquindox, and mequindox*.

*^b^Including higher than this tested MIC value*.

*^c^Including lower than this tested MIC value*.

*^d^Category I: antimicrobials of very high importance in human medicine, essential to the treatment of serious bacterial infections, no alternatives for resistant infections; Category II: antimicrobials of high importance in human medicine, used to treat a variety of infections, alternatives for resistance to category III antimicrobials; Category III: antimicrobials of medium importance in human medicine, used as first-line drugs, alternatives for resistance are generally available; Category IV: antimicrobials that are currently of limited use in human medicine*.

*^e^CLSI interpretative criteria for this bacterium/antimicrobial combination are not currently available. Reference R breakpoint of Ceftiofur*.

*^f^CLSI interpretative criteria for this bacterium/antimicrobial combination are not currently available. Isolates with an MIC ≥4 μg/mL were reported as resistant*.

*^g^CLSI interpretative criteria for this bacterium/antimicrobial combination are not currently available. Isolates with an MIC ≥16 μg/mL were reported as resistant*.

*^h^CLSI interpretative criteria for this bacterium/antimicrobial combination are not currently available. Isolates with an MIC ≥64 μg/mL were reported as resistant*.

When antibiotic resistance was analyzed according to animal species, the resistance rates of *Salmonella* were different for the majority of the drugs tested (Table 4). Excluding imipenem, resistance to the other 23 drugs ranged from 1.90 to 49.52% in chickens and 0.40 to 48.18% in pigs. In comparison, *Salmonella* from dairy cows showed resistance to 12 types of drug, and resistance rates ranged from 0.95 to 10.48%.

**Table 4:** **Antibiotic resistance of *Salmonella* isolated from different food-producing animals unit: %**.

**Drug**	**Total (*n* = 457)**	**Chicken (*n* = 105)**	**Pig (*n* = 247)**	**Dairy cow (*n* = 105)**
AMP	26.46	31.43	35.22	0.95
AMX	25.82	30.48	34.41	0.95
AMC	4.38	2.86	6.88	0
CRO	6.34	20.00	3.24	0
CEF	7.44	20.95	4.45	0.95
CEQ	5.25	15.24	3.24	0
IPM	0	0	0	0
ATM	0.67	1.90	0.40	0
GEN	19.69	17.14	29.15	0
AMK	1.53	6.67	0	0
TET	27.58	22.86	41.30	0
DOX	28.22	23.81	42.11	0
CIP	24.07	25.71	32.39	2.86
ENO	24.51	26.67	32.79	2.86
LEV	19.04	19.04	27.13	0
NA	39.17	46.67	48.18	10.48
SXT	39.61	49.52	47.77	10.48
CHL	24.94	26.67	34.41	0.95
FFC	20.13	20.95	27.94	0.95
FOS	12.04	6.67	19.43	0
PB	18.82	23.81	24.70	0
AZM	16.19	15.24	23.08	0.95
OLA	26.48	40.00	30.77	2.86
MEQ	25.38	40.95	28.34	2.86

*AMP, Ampicillin; AMX, Amoxicillin; AMC, Amoxicillin-clavulanic acid; CRO, Ceftriaxone; CEF, Ceftiofur; CEQ, Cefquinome; IPM, Imipenem; ATM, Aztreonam; GEN, Gentamicin; AMK, Amikacin; TET, Tetracycline; DOX, Doxycycline; CIP, Ciprofloxacin; ENO, Enrofloxacin; LEV, Levofloxacin; NAL, Nalidixic acid; SXT, Sulfamethoxazole-trimethoprim; CHL, Chloramphenicol; FFC, Florfenicol; FOS, Fosfomycin; PB, Polymyxin B; AZM, Azithromycin; OLA, Olaquindox; MEQ, Mequindox*.

In total, 159 strains (34.72%) exhibited varying degrees of multidrug resistance (MDR) (Table 5), defined as resistance to at least three different classes of antimicrobials. The *Salmonella* spp. that exhibited MDR consisted of 104 strains from pigs (including 15 and 89 strains from healthy and diseased pigs, respectively), 51 strains from healthy chickens, and 4 strains from healthy dairy cows. MDR isolates showed diverse resistance to different classes of antibiotics, but were most frequently resistant to penicillins, fluoroquinolones, and quinolones (each occurring 11 times). β-lactam/β-lactamase inhibitor combinations appeared with the lowest frequency (five times) in *Salmonella* strains with MDR.

**Table 5:** **The number of multidrug-resistance *Salmonella* strains identified in sampled animals unit: strain**.

**Resistance pattern**	**Chickens**	**Pigs**	**Dairy cows**
3	3	6	1
4	16	7	2
5	1	4	0
6	3	7	1
7	6	13	0
8	7	18	0
9	4	3	0
10	9	13	0
11	2	22	0
12	0	7	0
13	0	4	0
Total	51	104	4

Based on analysis of MIC values and CLSI, 25 of the 457 isolates (5.47%) were confirmed as ESBL producers, with three suspected strains (Table 6). The ESBL-producing strains were obtained from different animals and areas as follows: two strains from chickens in Hubei, 15 strains from chickens in Henan, and eight strains from diseased pigs. Because the differences in MIC values between CAZ, CAZ/C and/or CTX, and CTX/C were not =3-fold based on CLSI, three strains from diseased pigs were suspected to be ESBL-producing strains.

**Table 6:** **MICs used to verify ESBL-producing *Salmonella***.

**Sample code**	**Ceftazidime (μg/mL)**	**Ceftazidime-clavulanic acid (μg/mL)**	**Cefotaxime (μg/mL)**	**Cefotaxime-clavulanic acid (μg/mL)**	**Source**
147	2	0.5	64	<0.25	Chicken/Henan
151	4	0.5	>64	<0.25	Chicken/Henan
157	2	0.25	64	<0.25	Chicken/Henan
165	4	0.5	>64	<0.25	Chicken/Henan
171	4	0.5	>64	<0.25	Chicken/Henan
173	4	0.5	>64	<0.25	Chicken/Henan
174	4	0.5	>64	<0.25	Chicken/Henan
175	2	0.25	64	<0.25	Chicken/Henan
176	4	0.25	64	<0.25	Chicken/Henan
199	4	0.5	>64	<0.25	Chicken/Hubei
209	2	<0.25	32	<0.25	Chicken/Henan
211	2	<0.25	32	<0.25	Chicken/Henan
216	64	0.5	>64	<0.25	Chicken/Henan
217	2	<0.25	64	<0.25	Chicken/Henan
218	64	0.5	>64	<0.25	Chicken/Henan
264	2	0.5	32	<0.25	Chicken/Henan
268	128	2	32	1	Chicken/Hubei
4p	0.5	<0.25	16	<0.25	Pig/HAUvh
78p	0.5	<0.25	32	<0.25	Pig/HAUvh
127p	0.5	0.5	4	0.25	Pig/HAUvh
128p	0.5	0.5	64	4	Pig/HAUvh
151p	>128	128	16	8	Pig/HAUvh (suspected)
153p	4	0.5	64	<0.25	Pig/HAUvh
159p	4	0.5	>64	<0.25	Pig/HAUvh
167p	4	0.5	>64	<0.25	Pig/HAUvh
187p	>128	>128	16	8	Pig/HAUvh (suspected)
201p	128	64	>64	>64	Pig/HAUvh (suspected)
212p	0.5	0.5	64	0.5	Pig/HAUvh
212p	0.5	0.5	64	0.5	Pig/HAUvh

*HAUvh, Huazhong Agricultural University veterinary hospital*.

The *Salmonella* serotypes of isolates differed in terms of their sensitivity to drugs (Table 7). Among all drug-sensitive isolates (*n* = 188), 80 isolates were identified in 17 specific serotypes. Typhimurium (*n* = 28) was the major serotype. Isolates of *Salmonella* strains with MDR showed 26 specific serotypes and the major serotypes were Enteritidis and IIIb (both *n* = 10). All serotypes were resistant to nalidixic acid, but differences were observed in the resistance profiles of *Salmonella* serotypes that exhibited MDR. The most common resistance profiles in *Salmonella* IIIb (*n* = 10) and Enteritidis isolates (*n* = 10) were AMP-AMX-SXT-NAL and SXT-OLA-MEQ-NAL, respectively. The resistance profile was AMP-AMX-TET-DOX-ENO-SXT-CHL-NAL in Typhimurium (*n* = 6), TET-DOX-CIP-ENO-SXT-CHL-FFC-NAL in Schwabach (*n* = 4), and PB-OLA-MEQ-NAL in Kouka (*n* = 4).

**Table 7:** **Distribution of resistant *Salmonella* isolates according to serovars unit: strain**.

**Serogroup, serotype**	**Pan-susceptible**	**Intermediary**	**Number of antimicrobial classes in resistance pattern**						**Total**
			**1~2**	**3~4**	**5~6**	**7~8**	**9~10**	**11~13**	
B, Typhimurium	28	18	5	0	0	5	1	0	57
B, Agona	13	0	1	1	0	0	0	0	15
B, Derby	5	0	2	2	0	0	0	0	9
B, II	4	0	1	0	0	0	0	1	6
B, Kunduchi	4	0	0	0	0	0	0	0	4
B, Schwarzenground	0	0	0	0	0	1	1	1	3
B, Lagos	1	0	0	0	0	1	0	1	3
B, Agama	0	1	0	0	0	0	0	1	2
B, Fyris	2	0	0	0	0	0	0	0	2
B, Farsta	1	0	0	0	0	0	0	0	1
B, Gloucester	0	1	0	0	0	0	0	0	1
B, Kingston	1	0	0	0	0	0	0	0	1
B, Kubacha	0	0	0	0	0	1	0	0	1
B, Saintpaul	0	1	0	0	0	0	0	0	1
B, Stanley	0	0	0	0	0	0	1	0	1
B, Travis	0	0	0	0	0	0	1	0	1
B, Tumodi	0	0	1	0	0	0	0	0	1
B, Uppsala	0	0	0	0	0	0	1	0	1
C1, III b	7	0	5	5	1	0	2	2	22
C1, Typhisuis	6	0	1	0	0	0	0	0	7
C1, II	3	0	1	0	0	1	1	0	6
C1, Schwabach	0	0	1	0	0	3	1	0	5
C1, Irumu	1	0	0	0	0	1	0	1	3
C1, Kaduna	0	0	0	0	0	0	2	0	2
C1, Birkenhead	0	0	1	0	0	0	0	0	1
C1, Cotonou	0	0	0	0	0	1	0	0	1
C1, Hissar	0	0	0	0	1	0	0	0	1
C1, Paratyphic	1	0	0	0	0	0	0	0	1
C1, Thompson	1	0	0	0	0	0	0	0	1
C2-C3, Quiniela	1	0	0	0	0	0	0	0	1
D1, Enteritidis	0	0	0	7	2	1	0	0	10
D1, Berta	0	0	0	1	0	0	0	0	1
D1, II	0	0	0	0	0	1	0	0	1
D3, II	1	0	0	0	0	0	0	0	1
E1, Newlands	0	0	0	0	0	2	0	0	2
E1, Meleagridis	0	0	0	0	0	1	0	0	1
E1, Simi	0	0	0	0	0	0	1	0	1
E4, Gnesta	0	0	3	0	0	0	0	0	3
E4, Kouka	0	0	0	3	0	1	0	0	4
E4, Rideau	0	0	0	1	0	0	0	0	1
E4, Visby	0	0	1	0	0	0	0	0	1
A, Unidentified subtypes	0	0	0	0	0	1	1	1	3
B, Unidentified subtypes	43	23	6	2	4	12	2	3	95
C1, Unidentified subtypes	39	3	14	6	5	5	4	15	91
C2-C3, Unidentified subtypes	2	0	0	0	0	0	1	1	4
D1, Unidentified subtypes	0	0	0	1	0	2	1	0	4
E4, Unidentified subtypes	0	1	8	2	0	0	0	0	11
Unidentified subgroup	10	0	2	3	1	2	6	4	28
Without-self-curing	14	1	8	1	1	3	2	4	34
Total	188	49	61	35	15	45	29	35	457

## Discussion

The prevalence of *Salmonella* spp. in the center of China were in agreement with those obtained from Sichuan province farm animals (Li et al., 2013), but differed from other areas. The results were higher than in EU (European Food Safety Authority, 2014), where chicken 2.7%, pig 6.3%, cattle 2.4%, but were lower than in USA (National Antimicrobial Resistance Monitoring System, 2011), where chicken 52.56% ~ 47.95%, pig 10.34% ~ 8.79%, cattle 23.02% ~ 33.20%. Differences in isolation rates can be interpreted based on differences in region, sample types, collection seasons, culture methods, isolation methodologies, culture media, and local environmental conditions. For example, salmonellosis cases increased over the summer months, peaking in August and September, began decreasing thereafter (European Centre for Disease Prevention and Control, 2013). Therefore, a global perspective should ideally be adopted during sample collection, isolate separation, and, in particular, long-term monitoring.

The prevalence of *Salmonella* serotypes differed among animals and regions. Serogroup B and C1 were dominant, this result was consistent with in USA (Antunes et al., 2011). Serotype differ from other study. The dominant in chicken, pig, dairy cattle were Typhimurium and Enteritidis, IIIb and Typhimurium, Typhimurium and Agona in this study, respectively. The dominant serotype from chicken were Derby and Typhimurium in Sichuan province (Li et al., 2013), from pig were Enteritidis Indianain in Shandong province (Lai et al., 2014). The dominant from chicken, pig, cattle were Kentucky and Enteritidis, Adelaide and Johannesburg, Montevideo and Dublin in USA (National Antimicrobial Resistance Monitoring System, 2011); and from chicken, pig, cattle were Enteritidis and Infantis, Typhimurium and Typhimurium (monophasic), Typhimurium and Dublin in EU (European Food Safety Authority, 2014). This difference suggests serogroups and serotypes are varying according to geographical regions, and diversities and complexities. Certain serovars can emerge within a country or region for a certain period and then disappear with no evident cause or intervention. For instance, compared with data in 2010, the number of Enteritidis and Typhimurium decreased by 6% and 9% in 2011, respectively, (European Centre for Disease Prevention and Control, 2013); similar, compared with results in 2009, Enteritidis decreased, even disappeared from chicken and pig in 2012 from Shandong province (Lai et al., 2014).

The *Salmonella* serotypes isolated from farm animals overlapped with those that cause illnesses in humans, further highlighting the fact that *Salmonella* could be transmitted from animals to humans via the food chain (Alcaine et al., 2006; de Jong et al., 2009), such as Typhimurium and Enteritidis had identified from human in Henan and Hubei provinces (Cui et al., 2009; Xia et al., 2009). Some researchers have suggested that the differences in the dominant serotypes between animals and humans might be due to variations in their pathogenicity and corresponding resistance profiles (Volf et al., 2010), the strain containing Typhimurium and Enteritidis could acquired resistance to a large number of different antimicrobial compounds. At least two different serotypes existed from multiple-two strains to multiple-eleven strains, especially Enteritidis and Typhimurium, which were similar with other study from animals (Li et al., 2013; European Food Safety Authority, 2014; Lai et al., 2014), also with human (Cui et al., 2009; Xia et al., 2009). This may be related to the presence of the genetic structure known as *Salmonella* genomic island 1 (SGI1) and resistance genes (European Food Safety Authority, 2014). Therefore, monitoring of *Salmonella* should preferably focus more strongly on serovars that could, for example, be involved in a large outbreak.

Based on our MIC analysis, antibiotic-resistant bacteria, including multidrug-resistant bacteria, appeared in both healthy and diseased farm animals. These results were similar with other study (Gong et al., 2013; Li et al., 2013; Lai et al., 2014). One potential explanation is the use of antibiotics during breeding. Disease control and prevention in China during the breeding process mainly depends on the use of antibiotics (Gong et al., 2013). This arguably irrational use of antibiotics has contributed to the emergence of multidrug-resistant bacteria under selective antimicrobial pressure. Huazhong area (Henan, Hubei, and Hunan provinces) is one of the more concentrated areas of farm animals (China Agriculture Statistical Report, 2012), the demands and uses of drugs are huge, then inevitable there would be unreasonable use of drugs. These drugs, or the raw materials of the drugs, are inexpensive and easy to obtain; they have been widely used on animals in China. However, some steps have been taken to prevent the emergence of drug-resistant bacteria and promote food safety and public health with the Ministry of Agriculture of the People's Republic of China having formulated plans to ban or reduce the use of specific antimicrobials (Ministry of Agriculture of the People's Republic of China, 2002).

After the therapeutic effects of antimicrobials were confirmed in humans in the mid-1940s, they were soon introduced to veterinary medicine (McEwen, 2006). Even though some drugs were exclusively designed for veterinary use, the compounds administered are identical or very similar to those used in human medicine because they belong to the same antimicrobial classes (Heuer et al., 2009) such as β-lactams, cephalosporins, aminoglycosides, macrolides, tetracycline, sulfonamides, fluoroquinolones. Most of the antibiotic resistance to different classes has appeared in humans, animals, and/or animal products. Ceftriaxone had showed resistance rates ≤13% and 4% from imported foods and humans (Akiyama and Khan, 2012; Wong et al., 2014). Similar, gentamicin had shown resistance in animals and humans (Lai et al., 2014; Wong et al., 2014). Overlapping resistance not only can lead to treatment failure, but also have negative consequences in both humans and animals.

ESBL-producing *Salmonella* strains were isolated from chickens and pigs in different areas in this study. Currently, the resistance genes were still on attempting to identify and sequencing from the samples. The emergence of producing-ESBLs bacteria in animals and animal products is of particular concern to public health, it allow bacteria to become resistant to a wide variety of penicillins and cephalosporins. ESBL-producing bacteria have appeared in poultry, pig, and cattle farms (Horton et al., 2011; European Food Safety Authority, 2014). In *Salmonella*, acquisition of resistance genes was likely to have occurred by conjugation, usually with other Enterobacteriaceae through the transfer of plasmids (European Food Safety Authority, 2014). Bacteria that develop resistance via ESBLs could become a reservoir of resistance genes, which may enter the food chain (European Food Safety Authority, 2012). When antibiotic resistance occurs during infection, the remaining treatment option is usually an antibiotic from the carbapenem family. While carbapenems were previously drugs of last resort, their use is now also contributing to resistance (Centers for Disease Control and Prevention, 2014). They are used to treat highly resistant infections in humans, and are not used in food-producing animals. However, carbapenemase-producing organisms have been isolated from farm animals (Fischer et al., 2012). In China, monitoring strategies do not currently contain testing and identification of ESBL- or AmpC-producing organisms in isolates from animals and/or animal-derived food products. It suggest that this should be established within surveillance procedures to anticipate possible changes in the status of ESBL enzymes.

*Salmonella* isolates showed multiple-resistance in results, it suggested the emergence of co-resistance and/or cross-resistance (Cantón and Ruiz-Garbajosa, 2011). For example, amikacin had showed low levels of resistance (0.67%); this may be due to cross-resistance among aztreonam, ciprofloxacin, and β-lactams (Livermore, 2002). Co-resistance to fluoroquinolones and third- and fourth-generation cephalosporins has also been identified in *Salmonella* isolated from humans (Zhang et al., 2014). Considering these results, the prudent use of antibacterial agents should be strongly recommended in clinical, veterinary, and agricultural settings in order to preserve antibiotic activity and avoid the development of cross-resistance.

In previous studies, researchers have demonstrated the relationship between the increased prevalence of antimicrobial-resistant bacteria and (a) the increased use of antimicrobials in human and veterinary medicine, (b) greater movement of people and animals, and (c) increased industrialization (Cheng et al., 2012). Huazhong is central to China; this area not only meets the needs of its own people via the production of animals and food but also supplies other areas and imports animals and food from other regions. This movement is a potential contributor to the spread of antibiotic-resistant bacteria. The environment itself also contains a variety of bacteria that potentially represent an immense pool of antibiotic-resistance genes, which if transferred between bacteria could cause human and animal disease (D'Costa et al., 2006). For example, wild animals that are carriers of antimicrobial-resistant bacteria could pass on colonies of resistant strains when they encounter humans, farming areas, and/or waste (Literak et al., 2011). A limitation of our study was that the samples were only collected from farm animals. For complete consideration in a surveillance system, samples should also be collected from, for example, the farm environment and farm workers.

Despite bans and legislation, some antibiotics are still routinely fed to livestock prophylactically to increase profits and to limit potential bacterial infections in stressed and crowded livestock (Ndi and Barton, 2011). Antibiotic-resistant bacteria will inevitably follow wherever antimicrobials are used; therefore, a coordinated multi-disciplinary approach will be required to address this issue (Smith et al., 2009). Various programs around the world have been established to monitor and control antibiotic-resistant bacteria. Recently, an official document, “National Medium- and Long-Term Planning for Prevention and Control of Animal Epidemics (2012 to 2020),” (General Office of State Council of China, 2012) was issued in China. The government outlined plans to control diseases by detection and purification using a medium- or long-term program, with the aim of reducing the antimicrobials used in animals and in symptomatic treatment. In future, the safest way to prevent the problem may be reducing antimicrobial usage with strict policies and legislation to control the development and spread of resistant bacteria in animals.

## Funding

This research is supported by the Ministry of Science and Technology of the People's Republic of China (2012 BAK01B02), National Natural Science Foundation of China (31101856/31302143), Grants from National Basic Research program of China (2013CB127200), National Key Technology R&D Program (2012BAK01B00), Fundamental Research Funds for the Central Universities (2662015PY035) and project supported by the morning program of Wuhan in China (2015070404010191).

## Author contributions

XK, HH, MD, YW, and ZY designed the study. XK, HH, IA, and ZL collected the antimicrobial susceptibility data. XK, HH, YW, and ZL collected the serotype data. XK, HH, MD, and ZY analyzed the data. XK, HH, IA, and YW wrote the manuscript. XK, HH, MD, and ZY revised manuscript.

### Conflict of interest statement

The authors declare that the research was conducted in the absence of any commercial or financial relationships that could be construed as a potential conflict of interest.
